# People-centered tuberculosis care versus standard directly observed therapy: study protocol for a cluster randomized controlled trial

**DOI:** 10.1186/s13063-015-0802-2

**Published:** 2015-06-22

**Authors:** Vahe Khachadourian, Nune Truzyan, Arusyak Harutyunyan, Michael E. Thompson, Tsovinar Harutyunyan, Varduhi Petrosyan

**Affiliations:** School of Public Health, American University of Armenia, 40 Marshal Baghramyan Ave, Yerevan, 0019 Armenia; Department of Public Health Sciences, College of Health and Human Services, University of North Carolina at Charlotte, 9201 University City Boulevard, CHHS 427D, Charlotte, NC 28223-0001 USA

**Keywords:** tuberculosis, treatment adherence, innovative care, family support, psychological counseling, SMS reminder, cluster randomized trial

## Abstract

**Background:**

Tuberculosis is a major public health concern resulting in high rates of morbidity and mortality worldwide, particularly in low- and middle-income countries. Tuberculosis requires a long and intensive course of treatment. Thus, various approaches, including patient empowerment, education and counselling sessions, and involvement of family members and community workers, have been suggested for improving treatment adherence and outcome. The current randomized controlled trial aims to evaluate the effectiveness over usual care of an innovative multicomponent people-centered tuberculosis-care strategy in Armenia.

**Methods/design:**

Innovative Approach to Tuberculosis care in Armenia is an open-label, stratified cluster randomized controlled trial with two parallel arms. Tuberculosis outpatient centers are the clusters assigned to intervention and control arms. Drug-sensitive tuberculosis patients in the continuation phase of treatment in the intervention arm and their family members participate in a short educational and counselling session to raise their knowledge, decrease tuberculosis-related stigma, and enhance treatment adherence. Patients receive the required medications for one week during the weekly visits to the tuberculosis outpatient centers. Additionally, patients receive daily Short Message Service (SMS) reminders to take their medications and daily phone calls to assure adherence and monitoring of treatment potential side effects. Control-arm patients follow the World Health Organization - recommended directly observed treatment strategy, including daily visits to tuberculosis outpatient centers for drug-intake. The primary outcome is physician-reported treatment outcome. Patients’ knowledge, depression, quality of life, within-family tuberculosis-related stigma, family social support, and self-reported adherence to tuberculosis treatment are secondary outcomes.

**Discussion:**

Improved adherence and tuberculosis treatment outcomes can strengthen tuberculosis control and thereby forestall tuberculosis and multidrug resistant tuberculosis epidemics. Positive findings on effectiveness of this innovative tuberculosis treatment people-centered approach will support its adoption in countries with similar healthcare and economic profiles.

**Trial registration:**

ClinicalTrials.gov registration number: NCT02082340. Date of registration: 4 March 2014.

## Background

Tuberculosis (TB) is an airborne infectious disease transmitted from person to person, and is a major public health concern [1]. In 2012, TB affected more than 8.6 million people worldwide and resulted in 1.3 million deaths, which made it second only to HIV among leading causes of infectious disease-related deaths [1]. More than 80 % of active TB cases in the world are concentrated in 22 low- and middle-income countries [2], where 95 % of all TB deaths occur [3]. Moreover, TB is the leading cause of death among HIV patients, accounting for 20 % of all causes of deaths among HIV-infected people [2].

Drug-sensitive strains of TB are treated with four standard first-line anti-TB drugs. Non-compliance to treatment, misuse, or mismanagement of medications may lead to relapse, development of drug-resistant (DR) TB (which requires a more expensive and intensive treatment course), and even death [4, 5]. According to the 2013 World Health Organization (WHO) progress report [1], each year an estimated 450,000 cases and 150,000 deaths occur globally due to multidrug-resistant TB (MDR-TB). According to 2012 WHO data, 3.6 % of all new TB cases were MDR-TB, while the rates among previously treated patients reached 20 % [6]. Among those with MDR-TB, approximately 9.6 % suffer from extensively drug-resistant (XDR) strains. In addition to these adverse health consequences, TB places a tremendous economic burden on individuals and societies [7].

### Current treatment approaches and alternative strategies

Currently, WHO recommends directly observed therapy (DOT) to monitor patients’ adherence to the treatment regimen, with an optimal dosing frequency of 6 days per week for new TB patients. In DOT, patients take their medications while being observed by a healthcare worker, usually a physician or a nurse [8]. DOT prevents patients from missing their treatment and thus reduces the development of resistance. The outpatient phase of DOT, requiring near daily visits to the health facility, introduces significant financial and time-related costs to patients [9], particularly in areas with poor access to health services [10]. Given such limitations, researchers have implemented and evaluated various modified or alternative strategies to improve patients’ adherence to TB treatment [5, 11–14]. A systematic review of randomized controlled trials did not suggest enough evidence that DOT compared to self-administered treatment leads to better treatment outcomes in people receiving treatment in low-, middle-, and high-income countries [5].

Empowering patients is recommended as one of key components of TB treatment [15]. Although several authors have documented the effectiveness of patient empowerment in TB treatment [16–19], as identified in a review by Macq *et al.* in 2007 [20], people-centered solutions have received less attention globally. In low-resource countries, as an alternative to traditional DOT, researchers have recruited family members to observe patients’ medication intake, making treatment easier and less expensive [10, 21]. Family members are interested in identifying noncompliance and motivated to encourage and promote treatment adherence. Several studies [10, 21, 22] have documented the effectiveness of involving family members in TB treatment. Some patients value family involvement, especially if the observer is a family decision maker or is highly respected in the family [23]. Moreover, WHO has encouraged the development of innovative people-centered care strategies that, where medically appropriate, enable patient self-management and community engagement to improve health outcomes [24, 25].

In recent years, the widespread availability of mobile phones [26] has spawned numerous health communications interventions [27–29]. The Short Message Service (SMS) has been widely used as a reminder for patients to take their medications and improve treatment adherence [27–29]. Although, SMS reminders have shown significant improvement in medication adherence among patients with HIV [27], asthma [30], and other conditions, a recent review paper evaluating the effectiveness of SMS reminders for treatment adherence among TB patient was inconclusive, identifying methodological limitations of prior studies and suggesting further controlled trials evaluating the role of SMS reminders for TB adherence [31].

Patient counseling and education have been found to improve treatment adherence among TB patients [11, 32], which in turn, results in better treatment outcomes and improvement in the patients’ quality of life [33].

Treatment adherence has been associated with many different factors [9], including social support, healthcare satisfaction, and disease-related knowledge. Multicomponent interventions were found to improve treatment adherence [34]. Hence, our team developed a multicomponent innovative approach to TB care to enable self-administered drug intake by empowered TB patients - supervised by a trained family member and supported by patient and family counseling and reminders - to improve treatment adherence and outcome, and thereby forestall TB and multidrug resistant TB epidemics. The innovative treatment approach integrates several educational, technological, and social evidence-based components.

### Aims and objectives

The current randomized controlled trial aims to evaluate the effectiveness of an alternative multicomponent TB outpatient care strategy consisting of the following: 1) education and counseling for drug-sensitive TB patients and their family members, 2) self-administered drug-intake supervised by a trained family member, 3) daily SMS reminders to TB patients, and 4) daily phone calls to supporting family members. The research team is measuring if this alternative TB care strategy offers advantages over the regular DOT in terms of treatment outcomes, patients’ and family members’ knowledge about TB, patients’ depression status and quality of life, TB-related stigma, family social support, and self-reported adherence to TB treatment.

## Methods/design

### Trial design

This is an open-label, stratified cluster randomized controlled superiority trial with two parallel equal arms (intervention and control) conducted in Armenia. TB outpatient centers defined the clusters, with equal numbers of clusters assigned to intervention and control arms. We performed a cluster-level random assignment of drug sensitive TB patients to intervention and control arms to mitigate potential contamination of participants in the control arm.

### Study setting and eligibility criteria for clusters

The intervention takes place during the continuation phase of TB treatment and is implemented through Armenia’s TB outpatient centers. TB centers having a minimum patient load of five drug-sensitive TB patients in 2012 were included to ensure that centers with very small patient loads did not decrease the study’s power. Out of 60 TB outpatient centers in Armenia, 52 met the inclusion criterion.

### Eligibility criteria for individual tuberculosis patients

Study participants are patients starting their continuation phase of TB treatment in selected outpatient TB care centers. The patient eligibility criteria are as follows:

1. Having a diagnosis of drug sensitive pulmonary TB,
2. Starting the continuation phase of TB treatment between March and-December 2014,
3. Being at least 18 years old at the time of enrolment, and
4. The ability to communicate in Armenian.

The eligibility status of each patient is subject to verification by the corresponding TB outpatient center physician and the psychologist of the National Tuberculosis Control Center (NTC).

### Assignment of intervention/randomization

We stratified the TB outpatient centers (clusters) based on their drug-sensitive patient load in 2012 and their treatment outcomes (performance). We classified TB outpatient centers as small, average, or large, and performance outcomes as below average, average, or above average, yielding nine distinctive cluster categories. We applied computer-assisted block randomization to assure relatively equal number of cluster representatives from each of these nine categories in the intervention and control arms. The process yielded 26 centers in each arm.

### Cluster level interventions

TB physicians in both intervention and control clusters are instructed and requested to inform the study team whenever they have a patient who meets the inclusion criteria. In addition, all TB physicians and nurses in the intervention clusters are trained on the study protocols and procedures. They are instructed to closely follow the study protocols and perform study-related paper work, including the distribution and collection of weekly side effect and medication intake forms (applicable only to intervention clusters). Physicians in the intervention clusters also are trained to provide patients with a one-week supply (6 days of pills) of TB medications using the designated pillboxes.

### Individual level interventions

Patients in the intervention arm are asked to identify a family member to provide support during the course of treatment. The patients, their supportive family member, and other interested family members, as permitted by the patient, participate in TB educational and psychological counseling session provided by a team of a psychologist and a nurse (interventionists).

The interventionists participated in a training-of-trainers course before starting fieldwork. The interactive TB educational and psychological counseling session covers various topics, including TB symptoms, TB route of transmission, TB treatment strategies, importance of treatment adherence, prevention and infection control, handling side effects, addressing TB-related stigma and common myths. Average duration of each session is approximately 90 minutes. The primary objective is to empower TB patients and their family members to take responsibility for the treatment.

After the educational and counseling session, patients visit their physicians weekly in the TB outpatient centers for a medical check-up and to receive the prescribed TB medication for that week. The supportive family members are responsible for following the TB patients and supporting their adherence to the treatment regimen. The supportive family members also are responsible for completing a form each week documenting medication intake and observed side effects. Patients return the completed form to the TB physician during the next week’s visit.

Patients receive daily SMS messages each morning reminding them to take their medications on time, and weekly SMS messages reminding them to visit their TB physician for their weekly supply. The supportive family members receive daily phone calls from the research team each evening to inquire about side effects related to TB medication and to inquire if the patients are taking their TB medications on a timely basis. During these calls, patients and family members also are offered additional psychological advice and support if needed.

Patients in the intervention arm who lack a supporting family member also assume all the responsibilities defined for the supportive family member.

Patients in the control arm follow the regular DOT strategy for continuation phase, in line with the guidelines of Armenia’s Ministry of Health and WHO recommendations: it includes taking prescribed TB medication 6 days per week while being observed by a health-care provider. The patients in this group do not receive any additional intervention.

### Outcomes and additional measurements

The primary outcome of this controlled trial is TB treatment outcome according to WHO definitions as recorded in the medical file and reported to the NTC. Treatment outcomes are classified as follows: cured, completed treatment, dead, failed, defaulted, and transferred [35]. A patient is considered to be successfully treated if he/she has a cured or completed treatment outcome. All other categories are considered as an unsuccessful treatment outcome.

Secondary outcome measures include patients’ knowledge, depression status, quality of life, within-family TB-related stigma, family social support, and self-reported adherence to TB treatment. In addition, we measure family members’ knowledge, depression status, social support toward TB patient, and TB-related stigma. All the secondary outcomes other than self-reported treatment adherence are measured at T0 (baseline) and T1 (follow-up). Self-reported treatment adherence is assessed only at T1. The knowledge variable is constructed from correct responses to five items adapted from previous work [36–38]. Depression is assessed using a modified version of the Center for Epidemiological Studies Depression (CES-D) scale [39]; the Armenian translation is validated and the negatively formulated 16 items show high diagnostic accuracy and good factorial structure [40, 41]. We have used a modified version of a validated scale to measure within-family TB related stigma [42] and the Berlin social support scale [43] to measure social support of family members toward TB patients. We have applied EQ-5D to obtain data on quality of life of patients [44].

Measurements on sociodemographic characteristics including age, gender, socioeconomic status, education, marital status, and health behavior (smoking and drinking) are obtained to verify the validity of randomization and, if necessary, to control for the potentially confounding effects of these variables on the primary and secondary outcomes. Table 1 summarizes the study outcomes and presents the frequency of data collection.

**Table 1:** Outcome variables

			Timeline	
Variable	Source	Type	T0^a^	T1^b^
Treatment outcomes	National Tuberculosis Control Center reported treatment outcome evaluation	Categorical		X
Possible Depression	Patient survey	Continues	X	X
Quality of life	Patient survey	Continues	X	X
TB knowledge	Patient survey	Continues	X	X
TB-related stigma within family	Patient survey	Continues	X	X
Social support	Patient survey	Continues	X	X
Treatment adherence	Patient survey	Ordinal		X
Possible depression	Family member survey	Continues	X	X
TB knowledge	Family member survey	Continues	X	X
TB-related stigma	Family member survey	Continues	X	X
Social support	Family member survey	Continues	X	X

^a^At the beginning of continuation phase of treatment/before intervention

^b^After completion of continuation phase of TB treatment

*TB*, tuberculosis

Baseline and follow-up surveys are interviewer-administered. Given the characteristics of the intervention protocol, blinding interviewers assessing secondary outcomes (knowledge about TB, depression, quality of life, within-family TB-related stigma, and family social support) at baseline is not possible. However, interviewers are blinded for the assessment of secondary outcomes at follow-up. The treatment outcome (primary outcome variable of interest) is assessed and reported by the corresponding TB outpatient center physician as part of regular reporting to the NTC; this process is completely independent of the research team.

### Recruitment

The NTC informed physicians and nurses in all 52 eligible TB outpatient centers (clusters) of the study and invited them to be involved in the trial. All agreed to participate. Health-care workers in the intervention and control clusters are instructed and requested to report on all new and previously treated drug sensitive TB patients who start their continuation phase of TB treatment in the selected TB outpatient centers. The NTC psychologist, who had special training on the protocols and procedures of this study, makes the initial contact with the eligible patients following these steps: 1) the TB physicians report eligible patients to NTC, 2) the NTC psychologist contacts eligible patients over the phone, 3) the NTC psychologist verifies patients’ eligibility, 4) the NTC psychologist presents the study information briefly in a phone call and gets the patients’ consent to share their contact information with the research team, and 5) the NTC psychologist schedules a face-to-face meeting between the TB patient and the research team members.

The NTC psychologist also asks patients about household size and the number of family members aware of the TB diagnosis. If it becomes evident that all members of the patient’s family know about his/her disease and if the patient expresses desire for family members to receive TB counseling, then the NTC psychologist arranges an appropriate place (usually home of the patient), date and time for the counseling session. We ask the patients to identify potential supporters from their family members who participate in the counseling session to support them during their treatment period. If the patient lives alone or is unwilling for any family member to support him/her during the treatment and to participate in the counseling session, we set a convenient place (usually TB outpatient center), date and time for the TB patient’s counseling session.

Patients are enrolled within 15 days from the start of their continuation phase of treatment. The average duration of the continuation phase for the drug sensitive TB treatment is about four months. We anticipate completion of recruitment and data collection within 12 months from the start of the field work.

### Sample size

We based sample size calculations for this study on the treatment outcome as the main variable of interest. In 2012, 1,200 drug sensitive TB patients received the continuation phase of their treatment in TB outpatient centers. Utilizing the 2012 data on the number of patients and outcomes at each TB outpatient center, we identified 52 eligible clusters from the 60 centers. We calculated the intracluster correlation to be 0.04 and estimated the design effect as 1.6 [45]. The initial sample size calculation used a 10 % difference in the primary outcome [38], an alpha of 0.05 and power of 80 % and yielded a sample size of 95. Considering the potential for losses to follow-up taking a more conservative approach, we used a design effect of two; thus, the required sample size was calculated to be 190 in each arm.

### Data collection

We utilize interviewer-administered face-to-face surveys to collect data on secondary outcomes. All patients and their family supporters (if any) in the intervention and control arms are asked to complete an interviewer-administered questionnaire at baseline (T0) and after completion of their TB treatment (T1, follow-up).

### Data management

All the completed survey questionnaires are reviewed by a research team member for missing data and unusual responses. Data are double entered into an SPSS (version 21.0) database; after completing entry, the two databases will be reconciled to minimize data entry related errors.

The research team directly enters the data collected during daily calls made to the supporting family members of patients in the intervention arm into an Epi-info database.

The complete de-identified dataset of the trial will be publicly available when finalized.

### Statistical analyses

Statistical analyses will follow the intention-to-treat principle [46]. We will assess randomization across the two arms by comparing sociodemographic characteristics and other potential confounding variables using chi-square for binary and categorical variables, t-test, or other equivalent nonparametric tests, as appropriate, for continuous variables.

The primary outcome (treatment outcome) will be analyzed using generalized mixed effect model applying binomial distribution, logistic regression [47]. The status of randomization (intervention/control) will be included as an independent variable; to account for the clustered design we will treat clusters as a random effect. Potential confounding variables (age, gender, socioeconomic status, and others) with significant differences across intervention and control arms will be included as fixed factors. Secondary outcomes including knowledge, depression status, quality of life, within family TB-related stigma, and family social support also will be tested using generalized linear mixed effect models using logistic regression or linear regression, as appropriate. The secondary outcome model also will include its baseline assessment as a covariate.

We will report all the descriptive and inferential statistics results with their 95 % confidence intervals.

### Patient safety and monitoring of adverse events

TB patients included in this trial are drug-sensitive and have completed their intensive treatment phase. We take special efforts to minimize potential adverse events. All patients in the intervention arm undergo routine medical check-ups during weekly visits to TB outpatient centers. Moreover, the side effect report form is completed daily and reported by telephone to the research team, allowing the research team to quickly report concerns to the corresponding TB physician for follow-up. Side effects among those patients in the control arm are taken care during their regular daily visits to TB outpatient centers.

### Research ethics

The American University of Armenia Institutional Review Board (IRB) reviewed and approved the study protocol (# AUA-2014-020). Study-related activities started only after receiving IRB approval.

In this multicomponent intervention, we randomized at the TB outpatient center (cluster) level. Individual patient assignments therefore are determined by their geographically assigned TB outpatient center. Patients (and their family members) can only participate in the intervention arm after reading and signing a written consent form. Patients opting out of their assignment and those in the control arm receive traditional DOT for the continuous phase of TB treatment.

### Data safety

All patients are assigned a unique ID. The questionnaires and other data collection forms contain no identifiable information. Hardcopy questionnaires are stored in a locked safe in the research center at the American University of Armenia School of Public Health and will be archived for at least three years. Electronic databases are password protected and select members of the research team have access to the databases. No identifiable information is provided to persons outside the research team.

## Discussion

TB poses a major burden on populations, particularly among those living in low- and middle- income countries. Any improvement in TB treatment strategies can have a significant public health and economic importance. Improved control and management of TB can lead to better treatment outcomes, thereby decreasing health inequalities, morbidity, mortality, and economic costs associated with the disease. Rigorously evaluated alternative people-centered strategies, if successful, can be implemented in countries with similar healthcare and economic profiles and help to forestall TB and multidrug-resistant TB epidemics.

### Trial status

At the time of submission of this manuscript, the trial is ongoing; it began enrolment in March 2014.

## Abbreviations

CES-D: Center for Epidemiological Studies Depression
DOT: directly observed therapy
DR: drug resistant
IRB: institutional review board
MDR-TB: multi-drug resistant TB
NTC: National Tuberculosis Control Center
SMS: short message service
TB: tuberculosis
WHO: World Health Organization
XDR: extensively drug-resistant
